# How flexible is cognitive control? (Mouse) tracking conflict adaptation across context similarities

**DOI:** 10.1007/s00426-023-01874-0

**Published:** 2023-09-28

**Authors:** Hera Potamianou, Donna Bryce

**Affiliations:** 1 Department of Psychology, Eberhard Karls University of Tübingen, Schleichstrasse 4, 72076 Tübingen, Germany; 2 Department of Psychology, University of Augsburg, Augsburg, Germany

## Abstract

Exerting cognitive control to remain on-task and reach our goals is a crucial skill, as is the ability to flexibly adapt our responding in rapidly changing environments. The dynamics of cognitive control are typically studied by examining how participants process stimuli that contain competing relevant and irrelevant information in so-called conflict tasks. Adjustments in performance following the experience of conflict, also termed conflict adaptation, suggests a certain degree of flexibility in the deployment of cognitive control. The present study investigated to what extent conflict adaptation effects transfer across trials of the same and different tasks in three online mouse-tracking experiments. Adaptations of the Simon and Stroop tasks were combined to create different levels of context similarity between the paired tasks. Based on a previous review (Braem et al., Frontiers in Psychology 5:1–13, 2014), across-task conflict adaptation was expected only in the most and least similar contexts. In contrast to our hypothesis, conflict adaptation effects were observed in at least one measure in all three experiments. To our surprise, task order also seemed to impact the size of across-task conflict adaptation effects. The heterogeneity in the current results highlight the importance of using sensitive measurement tools to evaluate conflict adaptation and suggest that the occurrence of across-task conflict adaptation may be conditional on more than just shared relevant and irrelevant dimensions.

**Supplementary Information:**

The online version contains supplementary material available at 10.1007/s00426-023-01874-0.

## Introduction

Cognitive control constitutes an integral part of life in a society where one not only needs to follow rules and suppress impulsive behaviors in public environments, but also to ignore distractions and noise in one’s own private environment. “Noise” seems to gain more and more ground in the natural as well as the digital context, making numerous daily tasks such as navigating through a website while bombarded by visual and auditory advertisement an arduous journey of cognitive demands. To adapt and perform in a wide variety of contexts, cognitive control is required and recruited every step of the way. To study the capacity for cognitive control in the lab, real life situations that require selective attention towards a target while suppressing distractions are simulated through conflict tasks, also sometimes referred to as distracter interference tasks or stimulus response compatibility tasks (Eriksen & Eriksen, 1974; Simon & Rudell, 1967; Stroop, 1935). A key question in this line of research is to what extent and under which conditions cognitive control can be exerted effectively under changing contexts. In the current study, we examine the flexibility of cognitive control by studying how conflict adaptation effects transfer within and across different conflict tasks of varying similarity.

Conflict tasks aim to measure the level of engagement of cognitive control through the presentation of relevant information in combination with irrelevant information and require the participant to respond only to the target information. The relevant and irrelevant dimensions can activate the same or different response tendencies, giving rise to the so-called congruent and incongruent conditions of which the latter is thought to trigger the experience of conflict that is usually accompanied by more errors and longer reaction times compared to congruent conditions; a performance difference termed the congruency effect (Egner, 2007; Kornblum et al., 1990). Examples of classic conflict tasks are the Stroop (1935), Flanker (Eriksen & Eriksen, 1974) and Simon (Simon & Rudell, 1967) tasks. While these are often grouped together as conflict tasks, there is now widespread appreciation that they are distinct in many ways. For instance, the conflict arising in Simon tasks is traditionally associated with stimulus-response location, whereas in Stroop tasks it is associated with stimulus-stimulus dimensional overlap (Kornblum et al., 1999). Further, across the different tasks automatic and controlled processes are thought to unfold in different temporal scales (Ulrich et al., 2015), and the impact of relevant and irrelevant information appears to be distinct (Mackenzie et al., 2022). In the current study, this diversity in conflict tasks was exploited to create more and less similar task pairings and investigate the flexibility of cognitive control processes.

In addition to participants’ performance being affected by the current trial’s congruency, studies employing conflict tasks have reported sequential modulations of the size of the congruency effect depending on the previous trial’s congruency (e.g. Gratton et al., 1992). This more dynamic view of cognitive control has been termed conflict adaptation. More specifically, a reduced congruency effect following an incongruent trial, as compared to following a congruent trial, is a phenomenon now commonly referred to as the congruency sequence effect or Gratton effect (Braem et al., 2014; Egner, 2007). The congruency sequence effect (from here on referred to as the CSE) has been studied as a marker for conflict adaptation and was originally thought to arise because people expect the same type of trial to repeat and prepare for this (Gratton et al., 1992). Two types of CSE have been identified: the so-called within-task CSE (when the effect is observed across trials of the same conflict task) and the across-task CSE (when the effect is observed across trials of different conflict tasks). The latter is thought to reflect cognitive control transcending task context and as such reflects a surprising degree of flexibility of cognitive control.

A variety of theories and models have been suggested with respect to the source, underlying mechanism and specificity of conflict adaptation. The most prominent theories can be summarized in two groups according to their core concepts (Weissman et al., 2014): the ‘trial by trial modulation of cognitive control’ account and the ‘learning and memory account’. The former assumes the adaptive top-down allocation of attentional resources and examples of this category include the original proposition by Gratton et al. (1992) as well as the well-known conflict-monitoring theory (Botvinick et al., 2001). The learning and memory account assumes the effect arises because associations of different task features are learned and stored into memory and comprises theories such as feature integration (Hommel, 2004) and contingency learning (Schmidt & DeHouwer, 2011). While the different theoretical accounts disagree on the determinant factors and processes involved in the occurrence of a CSE, they offer a rich ground for research into the different mechanisms underlying conflict processing. It should, however, be noted that testing these different theoretical models is not the aim of the current study.

While observations of within-task CSEs have been reliably replicated across studies employing several variations of the classic conflict tasks, the findings for across-task CSEs are inconsistent and there remains debate regarding which shared or distinct features between tasks play a role in the presence of the CSE. In an effort to address this issue, Braem et al., (2014) suggested a framework in which a U-shaped function describes the relationship between the observation of an across-task CSE and context similarity between tasks. This function reflects that when two tasks can be identified as either highly similar or highly dissimilar with respect to their task sets, they are likely to lead to an across-task CSE. The reasoning behind this hypothesis, given that intuitively one may expect a linear rather than U-shaped relation, originates from whether the two contexts or features interfere with each other when they are both active in working memory. The assumption is that if the contexts differ to such an extent as to not interfere with each other, then both contexts can be maintained simultaneously in working memory. On the contrary, in the case of partial but not complete overlap between two contexts, interference will hinder co-activation of the two contexts, allowing for only one context to be available therefore inhibiting transfer effects between contexts. This framework is also in line with other conflict adaptation models and memory theories namely, theory of event codes (Hommel, 2004), task set level control theory (Hazeltine et al., 2011) and adaptation by binding theory (Verguts & Notebaert, 2009). The design of the current study was inspired by the U-shaped function proposed by Braem et al. (2014); we aimed to systematically manipulate the similarity of two tasks that were to be processed in alternation and examine to what extent conflict adaptation across tasks occurred.

### Response dynamics and mouse-tracking

In previous years, studies focusing on the mechanisms underlying decision-making assumed that the procedures of perception, programming a response, and executing a response constitute a linear sequence of events. A growing body of research, however, now proposes that these do not unfold sequentially and can influence each other throughout the decision-making process (Erb, 2018; Freeman et al., 2011; Gallivan et al., 2018). Studies employing both behavioral and neurophysiological methods have in fact shown that the same underlying system of neurons is responsible for sensory, cognitive and motoric components of the decision-making process and are simultaneously active during the process (e.g., Song & Nakayama, 2009). A considerable amount of literature employing mouse-tracking in a variety of domains including language (e.g., Spivey et al., 2005), social cognition (e.g., Freeman et al., 2008) and conflict processing (e.g., İkizoğlu & Çakır, 2021; Yamamoto et al., 2016; Ye & Damian, 2022) converge in finding that measures derived from the manual dynamics of a response, e.g., the mouse trajectories, are sensitive enough to capture small effects which often escape simple reaction time measures. In their study using Flanker, Simon and Spatial Stroop tasks, Ye and Damian (2022) found that trajectory measures as well as initiation times could sensitively capture previously experienced conflict. İkizoğlu and Çakır (2021) observed larger stimulus response compatibility effects in mouse-tracking measures than in response time measures when studying different versions of the Simon task. Employing a reverse Stroop task, Yamamoto et al. (2016) demonstrated that while interference effects were reflected in the response trajectories, facilitation effects were not. While conventional keyboard response measurements reflect the presence and ultimate resolution of conflict, mouse-tracking parameters can more directly capture the complexity of the cognitive processes underlying conflict; for example, how each task feature may contribute to response selection and at which temporal stage (Freeman, 2018; Hermens, 2018; Kieslich et al., 2020; Schoemann et al., 2020).

A variety of parameters have been analyzed in mouse- and other hand-tracking methods that are thought to provide additional insights into decision-making processes as they unfold. Three such movement variables, namely, the Movement Time (MT), Initiation Time (IT) and Maximum Absolute Deviation (MAD), are assessed in this study and will be presented here. The MT reflects the time interval between stimulus presentation and response completion and is therefore analogous to response times. The IT measures the time elapsed between the presentation of the stimuli to the start of the response movement and the MAD measures the degree of deviation of the cursor from the direct path to the final target (Wirth et al., 2020). Erb et al. (2016) propose that when measured in conflict tasks the IT and MAD reflect two processes, namely the response threshold adjustment process and the controlled selection process, respectively. The response threshold adjustment process refers to a temporary inhibition of all possible motor outputs in response to conflict. The controlled selection process has been suggested to reflect the ongoing competition between targets, with greater conflict resulting in greater attraction towards the incorrect response, translating into larger movement curvatures or degrees of deviation (Erb & Marcovitch, 2018, 2019; Erb et al., 2018). Notably, these mouse-tracking measures have primarily been studied within the context of a single conflict task. Here, we extend this approach to evaluate the sensitivity of these measures to detect across-task conflict adaptation effects.

### The current study

The aim of the present study is to investigate how flexibly cognitive control can adapt to different contexts. To this end, performance was examined in response to and following conflict in three experiments that varied the context similarity of the tasks employed. In each experiment a different pair of tasks was presented either alternating on a trial-by-trial basis in mixed blocks, or separately in single blocks. The different pairs aimed to establish gradual degrees of similarity with respect to relevant and irrelevant features. By manipulating the similarity between these dimensions, we can observe the extent to which changes in context impact conflict-induced adjustments in performance. Moreover, this study employed the dynamic response method of mouse-tracking, encompassing measures of both time and trajectory, which allowed for fine-grained observation of conflict processing and may inform future studies’ approach in evaluating conflict processes and resolution. The present study originates from a larger project studying the development of cognitive control; here the results of the adult age group are reported.

To assess performance adjustments in response to and following conflict, the presence of the CSE was assessed in the context of single tasks and across different tasks. Based on previous evidence (Hommel, 2004; Kerns et al., 2004; Notebaert et al., 2006; Wühr, 2005) we expected to observe within-task CSEs in all three experiments. Following up on Braem et al.’s (2014) proposed U-shaped function describing the relationship between context similarity and across-task CSEs, we expected to observe across-task CSEs in MT only in the case of very high (Experiment 1) and very low (Experiment 3) context similarity between tasks. Given that the majority of conflict adaptation studies have utilized keypress responses, there was less evidence on which to base our hypotheses of mouse-tracking measures. However, based on Erb and Marcovitch (2019) we anticipated the IT to be only affected by the current and previous trial’s congruency, but CSEs to also be reflected in the MAD.

## Experiment 1 (Animal Simon–Arrow Simon)

In Experiment 1, two versions of a (visual) directional Simon task were combined which had both relevant and irrelevant dimensions in common. The tasks only differed in their stimulus sets. As such, this experiment was considered to create a high context similarity between tasks.

### Methods

#### Preregistration, stimuli and data

This study’s preregistration document, stimuli and data can be found here: https://osf.io/v573g/. 

#### Participants

For each experiment reported here, a minimum sample size of *N* = 30 was selected. This minimum sample size was estimated based on a power analysis to detect an interaction effect (the CSE) of 40 ms (run using the script provided in section 5.3.2 of Wickelmaier, 2021). The effect size used in the power analysis was half the size of the within-task CSE observed in a pilot study of the Animal Simon task; this was deemed appropriate given that across-task effects may be expected to be smaller than within-task effects. Our simulations indicated that a sample size of *N *= 30 would have 87% power to observe this smaller effect size. Accordingly, 30 German speaking adults (*M*_age_ = 27.26, *SD* = 8.82, 21 female and 9 male) participated in Experiment 1. All participants apart from one were right-handed and the ratios of their highest educational levels were as follows: 47% Medium-High secondary education, 43% University degree, 7% Apprenticeship and 3% Low Middle secondary education. For each experiment reported, participants were recruited via social media and in public spaces via flyers and posters, participation was voluntary and compensated either with gift vouchers or course credits for University students. All procedures were approved by the Commission for Ethics in Psychological Research of the University of Tuebingen and all participants provided informed consent.

#### Materials and apparatus

Data were collected in 2021–2022. An online questionnaire including experimental instructions, demographic data and informed consent was generated using SoSci Survey (Leiner, 2019) and was made available to users via https://www.soscisurvey.de/. The experiment was designed and generated using PsychoPy 3, version: 2021.2.0 (Peirce et al., 2019) and was then hosted and distributed online via the Pavlovia (Bridges et al., 2020) platform. Participants were instructed to perform the experiment in a quiet, private environment on a laptop or desktop-computer equipped with a computer mouse. Information with respect to the screen and window size and resolution were collected to confirm participants had not used a cellphone or tablet to complete the experiment.

All experimental stimuli were displayed on a white background. All item sizes are described as height units as suggested by PsychoPy software for the implementation of online experiments to facilitate the scaling in any computer screen. Height units allow for adjustment with the window’s size, for example a variable of 0.4 size in height units corresponds to a stimulus presented as 40% of the window height. All item locations on screen are described as coordinates; as specified by PsychoPy software, the center of the screen is represented by coordinates (0,0), negative values indicate downwards/leftwards locations, and positive values indicate upwards/rightwards locations. Similar to stimulus size, location is also measured in height units, hence also referring to a percentage of the window’s height with respect to the distance from the screen’s center. After appearing on screen all items remained until the participant had concluded their action, unless specified otherwise.

Since these experiments were part of a larger developmental project designed to compare between children and adults, the conflict tasks used across the different populations were child-friendly versions of classic conflict tasks that did not rely on complex response rules or reading automatization. The trial stimuli for the Animal Simon and Arrow Simon tasks consisted of colored pictures of animals and arrows, respectively. Twelve unique animal images and 12 unique arrow images were included, each in both a left and right facing/pointing orientation, resulting in 24 stimuli for each task. In each trial, one stimulus was presented with the size set to 20% of the window’s height, and a horizontal distance from the center measuring 50% of the height of the window. Responses were made by moving the computer mouse and using either the left or right index finger (according to the handedness of the participant) for clicking. To facilitate the localization of the cursor on screen at every moment, a red dot was presented at the position of the cursor with size 2.5% the height of the window.

**Fig. 1:**
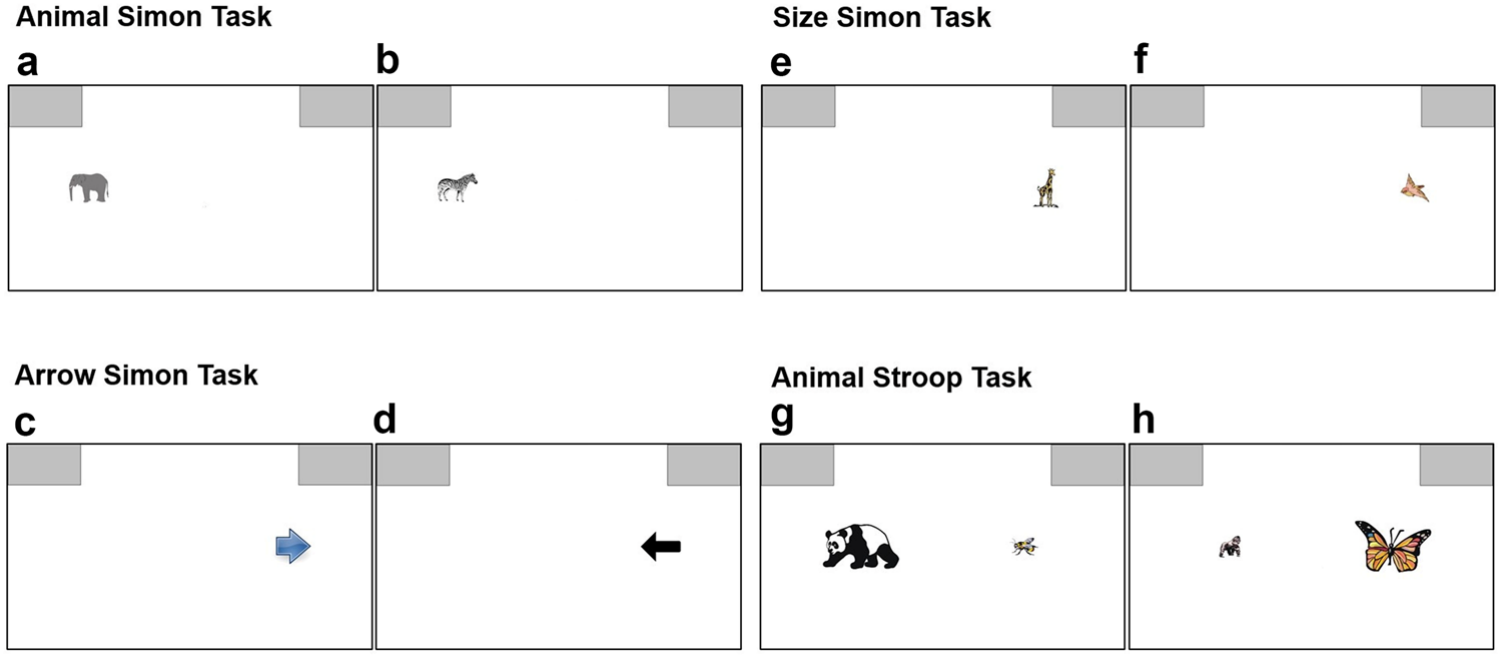
Illustration of congruent (**A**, **C**, **E**, **G**) and Incongruent (**B**, **D**, **F**, **H**) trials from each task employed across Experiments 1–3

An orange box of 10% the height of the window served as the starting point within which participants had to position their cursor in order for the trial stimulus to appear. A prompt in the form of an arrow measuring 20% of the window’s height appeared directly above this start-box in case of delayed movement initiation. The target areas for responding were presented as gray boxes of width that measured as 1/5 of window width and height that measured as 1/5 of the window height. These response-boxes were positioned in the top left and top right corners of the screen (see Fig. 1).

#### Procedure

The conflict tasks that were used in this experiment, namely the Arrow Simon and Animal Simon task, are both child-friendly versions of the directional Simon task. The stimuli were presented one at a time, either on the left or right side of the screen, facing either the left or the right direction. As the purpose of the task (see below) dictated, the location of the animal or arrow stimulus necessarily varied across trials. The participant’s task was to identify which direction the animal (arrow) was facing (pointing; relevant dimension), ignoring which side of the screen the animal (arrow) appeared (irrelevant dimension). In congruent trials (see Fig. 1A and C), the direction the animal (arrow) was facing (pointing) and the side on which it was presented were compatible; in incongruent trials (see Fig. 1B and D) they were incompatible. The Animal Simon task employed in this study was piloted in a separate experiment (*N *= 197, with button responses) where both a significant congruency effect, *F*(1, 196) = 35.47, *p* < 0.001, *η*_p_^2^ = 0.15, and a significant CSE,* F*(1, 196) = 326.84, *p* < 0.001, *η*_p_^2^ = 0.63, in reaction times were observed.

Prior to the experimental blocks, participants completed a block of calibration trials and practice trials. In the calibration trials the participants moved their cursor directly from the start-box to one of the response-boxes. The response-box appeared in the first eight calibration trials at the top left of the screen and the next 8 calibration trials at the top right of the screen. There were no trial stimuli included in the calibration trials and they served as familiarization trials. In 12 practice trials, participants processed trials that simulated the experimental trials and were provided positive or negative feedback (green “thumbs up” or red “thumbs down” icons). The positive feedback remained on the screen for 0.5 s, whereas the negative feedback remained on the screen for 2.5 s. Practice trials consisted of four Arrow Simon trials, four Animal Simon trials, and four trials that alternated between Arrow Simon and Animal Simon trials.

At the start of each trial (including calibration, practice and experimental) a fixation-eye was presented in the center of the screen, accompanied by the start-box at the center bottom of the screen. To start each trial participants had to place the cursor within the start-box. If the cursor positioning had not taken place within 2 s, a prompt in the form of an arrow pointing downwards at the start-box appeared directly above it. Once the cursor had been positioned within the start-box area for 300 ms, the trial stimulus and response-boxes appeared and remained on screen until the participant clicked on one of them and concluded the trial.

The participants were instructed to respond as quickly and accurately as possible to each trial by moving their mouse from the start-box to their selected response-box and clicking. The structure of the experiment was as follows: first the participants were presented with 16 calibration trials (8 for each side), followed by a block of 12 practice trials (four for each type of experimental block) and finally nine experimental blocks. The three types of experimental block (a single Arrow Simon block, a single Animal Simon block and an alternating block) were each repeated three times within the experiment in quasi-random order. The single Animal Simon block comprised 48 trials, the single Arrow Simon block comprised 48 trials, and the alternating block comprised trials of the Animal Simon and Arrow Simon tasks presented in an alternating fashion (in total 96 trials). The participants were able to take one break between each block (i.e., every 48 trials) and two breaks within the alternating tasks block (i.e. every 32 trials). Each block was balanced for congruent/incongruent trials as well as left- and right-target responses. The total duration of the experiment was approximately 30 mins.

Within the experiment, the following dependent variables were collected for each trial: response accuracy, response time, the x- and y-coordinate of the cursor on the screen and the time of each frame of the trial. The independent variable congruency of current trial was manipulated within-participants. On each trial, a left- or right-facing animal image was randomly selected without direct repetition from the relevant animal stimuli for the Animal Simon task, and a left- or right-pointing arrow image was selected without direct repetition from the relevant arrow stimuli for the Arrow Simon task. The parameters pointing/facing side (left/right) and presentation side (left/right) were combined to produce four unique Arrow Simon trials and four unique Animal Simon trials belonging to two Congruency conditions (congruent/incongruent). Within a block of 48 trials of one task, each unique trial was repeated 12 times and presented in random order.

#### Data analysis

##### Data exclusion

According to our pre-registration, a participant’s whole dataset would be excluded if they had an overall accuracy level of less than 60%; in this experiment no participant met that criterion for exclusion. The first trial of each block as well as practice trials were not analyzed. For analysis of accuracy, trials in which the previous trial was an error were excluded as they may have been affected by post-error slowing (0.28%), as were trials with extremely long intertrial intervals (ITIs; > 5 s) as they were considered to reflect trials in which the participant took a break (0.09%). For analysis of MT, IT and MAD, additionally to the previous exclusions, trials in which the current trial was an error were excluded (0.24%). Extremely long MTs, that is > 3 SD above the mean (calculated per participant and per experimental condition) were excluded, as they were considered to reflect trials in which the participant was distracted (1.92%).

##### Derived variables

In each experiment, the following response dynamics measures were derived from the mouse trajectories for each trial: initiation time (IT; that is, the time elapsed between stimulus presentation and movement initiation), and maximum absolute deviation (MAD; that is, the trajectory’s maximum deviation from a line directly connecting the start and final position of the mouse cursor). Before computing these two measures, all trajectories extracted from correct trials were remapped on the same side (left) and subjected to time-normalization to ensure an equal number of cursor position coordinates for all trajectories. The movement initiation was defined as a movement of the cursor of more than half the height of the start-box. Both measures were aggregated across trials per participant and condition.

##### Planned analyses

Data from each experiment were processed and analyzed using R (R Core Team, 2019) and mouse-tracking data was analyzed using the Mousetrap package in R (Kieslich & Henninger, 2017). Data from the single Arrow Simon block were analyzed to test for the presence of a within-task Arrow Simon CSE, data from the single Animal Simon block were used to test for a within-task Animal Simon CSE, and data from the alternating block were analyzed to test for two across-task CSEs (Arrow Simon-Animal Simon and Animal Simon-Arrow Simon).

The presence of conflict adaptation (the CSE) was evaluated via repeated measures ANOVAs on behavioral data measures (accuracy, MT) and response dynamics measures (IT, MAD). An interaction of the two within-subject factors current trial congruency (named congruency) and previous trial congruency (named *n-1* congruency) indicates the presence of a CSE.

##### Results and discussion

**Fig. 2:**
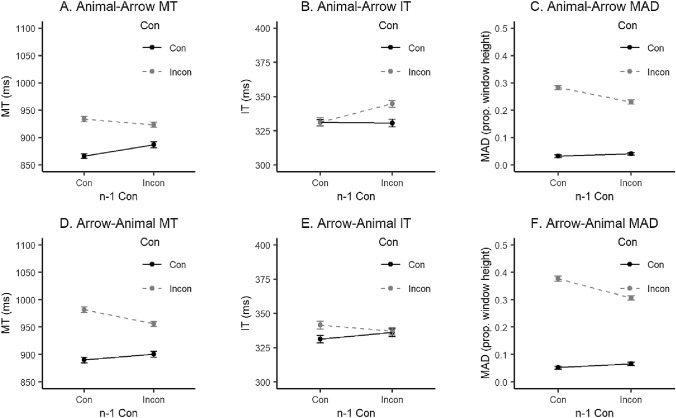
Across-task mean MT, IT and MAD for Experiment 1. In the upper panels (A, B, C) are the results for Animal Simon trials followed by Arrow Simon trials, in the lower panels (D, E, F) are the results for Arrow Simon trials followed by Animal Simon trials. *MT* movement time, *IT* initiation time, *MAD* maximum absolute deviation, *Con* congruency,* n−1* *Con* previous trial’s congruency. Error bars represent within-subject standard errors (using the method from Morey, 2008)

Statistical results from the repeated measures ANOVAs conducted on accuracy, MT, IT and MAD for both within-task and across-task analyses can be found in Table 1. For the Animal Simon task, a main effect of current trial congruency, herewith referred to as a congruency effect (CE), was observed in the MT, IT, and MAD. An additional significant interaction of the current trial’s congruency and the previous trial’s congruency, herewith referred to as a congruency sequence effect (CSE), was observed only in MAD values. In the Arrow Simon task, a CE was reflected in all measures and a CSE in MT and MAD values. Plots of the within-task results are presented in the Supplementary Materials, Fig. S1. For the across-task results see Fig. 2. When Animal Simon trials were followed by Arrow Simon trials, a CE was found in IT and MAD values, the latter of which also yielded significant results for the presence of a CSE. Lastly, when Arrow Simon trials were followed by Animal Simon trials, a CE was observed in MT, IT and MAD measures and a CSE in both MT and MAD values.

**Table 1:** Two-way ANOVA statistics for Experiment 1 study variables

Variable	Effect					
	Con		n−1 Con		Con × n−1 Con	
	*F*(1, 29)	*η*_p_^2^	*F*(1, 29)	*η*_p_^2^	*F*(1, 29)	*η*_p_^2^
Animal Simon within-task results						
ACC	3.81	0.12	0.20	0.01	2.11	0.07
MT	55.76***	0.66	0.00	0.00	3.40	0.10
IT	19.99***	0.41	15.81***	0.35	1.72	0.06
MAD	115.10***	0.80	5.91*	0.17	20.24***	0.41
Arrow Simon within-task results						
ACC	4.24*	0.13	2.97	0.09	0.01	< 0.01
MT	27.29***	0.48	2.63	0.08	5.02*	0.15
IT	4.58*	0.14	5.02*	0.15	0.08	< 0.01
MAD	91.62***	0.76	18.01***	0.38	14.43**	0.33
Animal Simon–Arrow Simon across-task results						
ACC	1.40	0.05	0.19	0.01	0.49	0.02
MT	48.78	0.63	0.97	0.03	3.00	0.09
IT	9.77**	0.25	8.02**	0.22	3.48	0.11
MAD	78.04***	0.73	8.29**	0.22	11.83**	0.29
Arrow Simon–Animal Simon across-task results						
ACC	4.11	0.12	0.77	0.03	1.18	0.04
MT	58.56***	0.67	1.92	0.06	30.54***	0.51
IT	5.34*	0.16	0.03	< 0.01	3.68	0.11
MAD	127.60***	0.81	24.69***	0.46	25.95***	0.47

*N* = 30. *ANOVA* analysis of variance; *ACC* accuracy, *MT* movement time, *IT* initiation time, *MAD* maximum absolute deviation, *Con* congruency, *n−1 Con* previous trial’s congruency

*p < 0.05

**p < 0.01

***p < 0.001

The aim of Experiment 1 was to create an environment with two alternating tasks that shared everything (instruction, relevant dimension, irrelevant dimension) except the stimulus features (arrow stimuli in the case of Arrow Simon and animal stimuli in the case of Animal Simon). Inspired by Braem et al.’s (2014) proposed U-shaped model describing the relationship between across-task CSEs and context dissimilarity, we expected to observe across-task CSEs in this experiment. The presence of an across-task CSE in MT in the case of Arrow Simon followed by Animal Simon trials is in line with our hypothesis. Furthermore, with respect to our additional mouse-tracking measures, MAD values seem to have captured across-task CSEs in both task orders. ITs yielded significant main effects for current and previous trial’s congruency which provides support for Erb et al.’s (2016) suggestion that this measure reflects a so-called response threshold adjustment process.

Notably, some of the effects appear to differ depending on task order, particularly in IT. This unexpected finding was further investigated in additional analyses. Trials from the alternating block were analyzed in a repeated measures ANOVA with the within-subjects factors congruency, n-1 congruency, and task order (Animal Simon–Arrow Simon vs. Arrow Simon–Animal Simon). Of particular interest is the three-way interaction, which would indicate that the size of the CSE is affected by task order. In this experiment, this three-way interaction reached significance only for the IT measure, *F*(1, 29) = 4.48, *p* = 0.043, *η*_p_^2^ = 0.13.

## Experiment 2 (Size Simon-Arrow Simon)

In Experiment 2 two versions of a visual Simon task were combined which had only the irrelevant dimension in common. The tasks differed in their relevant dimensions and stimulus sets. As such, this experiment was considered to create a medium context similarity between tasks.

### Methods

#### Preregistration, stimuli and data

This study’s preregistration document, stimuli and data can be found here: https://osf.io/jaunp/. 

#### Participants

A total of 35 (*M*_age_ = 25.14, *SD* = 6.47, 24 female and 11 male) German-speaking adults participated in this experiment. Participants’ ratios with respect to handedness were as follows, 91.43% right-handed, 5.72% left-handed, 2.85% ambidextrous, and with respect to highest educational level: 57.14% medium-high secondary education, 37.14% university degree, 5.72% apprenticeship and 0% low middle secondary education.

#### Materials and apparatus

The materials and apparatus were identical to those employed in Experiment 1. All experimental stimuli, excluding the trial stimuli related to the Size Simon task, were identical to Experiment 1. Stimuli for the Size Simon task consisted of 24 colored pictures of animals, taken from the Animal Stroop task developed by Bryce et al. (2011), specifically from the categories big and small (a distinction based on their real-life size). In each trial, one animal image sized 20% of the window’s height was presented either on the left or on the right side of the screen with a distance from the center measuring 50% of the height of the window. The trial stimuli for the Arrow Simon task were the ones used in the Arrow Simon task in Experiment 1.

#### Procedure

The conflict tasks employed in this experiment were the Size Simon task and the Arrow Simon task. The procedure was identical to that of Experiment 1, except that the Size Simon task was presented in the place of the Animal Simon task. The Size Simon task involved the presentation of one colored picture of an animal either on the left or on the right side of the screen. The participant’s task was to identify whether the animal is big or small in real-life (relevant dimension); participants were instructed to click the left-box for a small animal and the right-box for a big animal. The irrelevant dimension that they should ignore is the side of the screen on which the animal is presented. The Size Simon task was piloted in a separate experiment (*N*=27, with button responses) where both a significant congruency effect, *F*(1, 26) = 8.00, *p* = 0.009, *η*_p_^2^ = 0.24, and a significant CSE,* F*(1, 26) = 23.32, *p* < 0.001, *η*_p_^2^ = 0.47, in reaction times were observed.

In Fig. 1E and F an example of a congruent and incongruent trial of the Size Simon task is depicted. In a congruent trial, a small (large) animal was presented on the left (right) side of the screen and the correct response would be on the left (right). In an incongruent trial, a small (large) animal was presented on the right (left) side of the screen but the correct response would be on the left (right).

The experimental design and structure were identical to those of Experiment 1, as were the dependent and independent variables. On each trial, relevant stimuli were randomly selected without direct repetition from a pool of small or big animals for the Size Simon task, and from a pool of right- or left-pointing arrows for the Arrow Simon task. Each combination of parameters, namely correct response-side (left/right) and stimulus category (big/small-size for the Size Simon task, left/right-pointing arrows for the Arrow Simon task), was equally represented through four unique Size Simon trials and four unique Arrow Simon trials, creating two congruent and two incongruent trial types. Within a block of 48 trials of one task, each unique trial was repeated 12 times and presented in random order.

#### Data analysis

##### Data exclusion

Two participant’s whole datasets were excluded because they had an overall accuracy level of less than 60% in one of the two tasks. Data from *N *= 33 remained in the analysis. As for Experiment 1, the first trial of each block as well as practice trials were not analyzed. For analysis of accuracy, trials in which the previous trial was an error (3.96%) and trials with extremely long ITIs (0.11%) were excluded. For analysis of MT, IT and MAD, additionally to previous exclusions, trials in which the current trial was an error were excluded (0.38%). Extremely long MTs, that is > 3 *SD* above the mean (calculated per participant and per experimental condition) were excluded, as they were considered to reflect trials in which the participant was distracted (1.84%).

##### Planned analyses

Similar to Experiment 1, data from the single Size Simon blocks were analyzed to test for the presence of within-task Size Simon CSE, data from the single Arrow Simon blocks were analyzed for the within-task Arrow Simon CSE, and data from the alternating blocks were analyzed to test for two across-task CSEs (Size Simon-Arrow Simon and Arrow Simon-Size Simon). The presence of within-task and across-task conflict adaptation was evaluated following the same procedure as that in Experiment 1.

##### Results and discussion

**Fig. 3:**
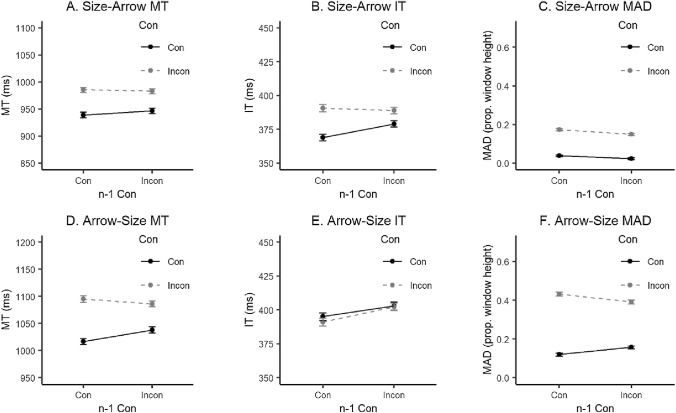
Across-task mean MT, IT and MAD for Experiment 2. In the upper panels (A, B, C) are the results for Size Simon trials followed by Arrow Simon trials, in the lower panels (D, E, F) are the results for Arrow Simon trials followed by Size Simon trials. *MT* movement time; *IT* initiation time; *MAD* maximum absolute deviation; *Con* congruency; *n-1 Con* previous trial’s congruency. Error bars represent within-subject standard errors (using the method from Morey, 2008). Responses were overall slower when the current task was the Size Simon task, reflected in different absolute values in y-axes for the MT plots

Statistical results from the repeated measures ANOVAs conducted on accuracy, MT, IT and MAD for both within-task and across-task analyses can be found in Table 2. Plots of the within-task results are presented in the Supplementary Materials, Fig. S2. For the within-task Size Simon results, a CE was observed in Accuracy, MT and MAD values. An additional interaction effect between current trial congruency and previous trial congruency (i.e. the CSE) was observed in MT and MAD values. In the Arrow Simon task, a CE was observed in MT, IT, and MAD and a CSE in MT and MAD values. The across-task data patterns are plotted in Fig. 3. When Size Simon trials were followed by Arrow Simon trials, a CE was found once again in all measures except accuracy and surprisingly a CSE was found for IT. Lastly, when Arrow Simon trials were followed by Size Simon trials, a CE was observed in MT and MAD values and a CSE in Accuracy, MT and MAD.

**Table 2:** Two-way ANOVA statistics for Experiment 2 study variables

Variable	Effect					
	Con		n−1 Con		Con × n−1 Con	
	*F*(1, 32)	*η*_p_^2^	*F*(1, 32)	*η*_p_^2^	*F*(1, 32)	*η*_p_^2^
Size Simon within-task results						
ACC	5.47*	0.15	0.00	< 0.01	0.48	0.01
MT	41.32***	0.56	1.91	0.06	15.55***	0.33
IT	4.12	0.11	17.35***	0.35	0.93	0.03
MAD	84.90***	0.73	12.52**	0.28	40.26***	0.56
Arrow Simon within-task results						
ACC	3.90	0.11	1.21	0.04	1.21	0.04
MT	88.87***	0.74	0.50	0.02	8.40**	0.21
IT	12.22***	0.28	5.18*	0.14	1.46	0.04
MAD	116.14***	0.78	36.23***	0.53	55.61***	0.63
Size Simon–Arrow Simon across-task results						
ACC	3.01	0.09	0.03	< 0.01	1.75	0.05
MT	45.10***	0.58	0.28	0.01	1.30	0.04
IT	24.81***	0.44	2.96	0.08	4.28*	0.12
MAD	44.21***	0.58	4.36*	0.12	2.05	0.06
Arrow Simon–Size Simon across-task results						
ACC	3.67	0.10	2.06	0.06	8.28**	0.21
MT	52.68***	0.62	0.46	0.01	7.99**	0.20
IT	0.63	0.02	22.61***	0.41	0.63	0.02
MAD	115.49***	0.78	1.68	0.05	22.30***	0.41

*N* = 33. *ANOVA* analysis of variance, *ACC* accuracy, *MT* movement time, *IT* initiation time, *Con* congruency, *MAD* maximum absolute deviation, *n−1* *Con* previous trial’s congruency

*p < 0.05

**p < 0.01

***p < 0.001

In contrast to Experiment 1, the aim for Experiment 2 was to create an environment where the two alternating tasks shared only the irrelevant dimension (the stimulus location on the screen) while the relevant dimension, instructions and stimulus features are distinctly different. Following Braem et al.’s (2014) model, in the case of only partial overlap in context similarity between tasks we hypothesized no across-task CSE. In contrast to our hypothesis, the across-task results from Experiment 2 (Table 2, Fig. 3), showed a significant CSE in three out of four measures in the case of Arrow Simon trials followed by Size Simon trials. Furthermore, and to our surprise, the mouse-tracking measure IT captured an across-task CSE in the case of Size Simon trials followed by Arrow Simon. When examining the data patterns in Fig. 3, one could speculate that there is a certain trade-off between the mouse-tracking measures. That is, when the impact of previous conflict (the CSE) emerges in the IT, it does not emerge in the MAD values (compare Fig. 3B and C), and when it is not present in the IT, the CSE is present in the MAD values (compare Fig. 3E and F). Consistent with previous findings, significant main effects for congruency and previous congruency were also observed in IT. As in Experiment 1, task order was further investigated in a follow up ANOVA including task order (Size Simon–Arrow Simon vs. Arrow Simon–Size Simon) as a factor. In this experiment, the three-way interaction reached significance for Accuracy, *F*(1, 32) = 14.96, *p* = 0.001, *η*_p_^2^ = 0.32, and for MAD, *F*(1, 32) = 14.68, *p* = 0.001, *η*_p_^2^ = 0.31.

## Experiment 3 (Animal Simon-Animal Stroop)

In Experiment 3, two tasks were combined (a Simon task and a Stroop task) which had neither the relevant nor the irrelevant dimension in common. The tasks also differed in their stimulus sets. While both tasks employed animal images as stimuli, it should be noted these were distinct and differed in style. As such, this experiment was considered to create a low context similarity between tasks.

### Methods

#### Preregistration, stimuli and data

This study’s preregistration document, stimuli and data can be found here: https://osf.io/pmhqg/. 

#### Participants

A total of 30 (*M*_age_ = 24.53, *SD* = 6.31, 17 female and 13 male) German-speaking adults completed the experiment. The participants’ handedness and highest education level ratios are presented here, 87% right-handed, 10% left-handed, 3% both-handed and in terms of education level category 57% medium–high secondary education, 33% university degree, 7% apprenticeship and 3% low middle secondary education.

#### Materials and apparatus

The materials and apparatus were identical to those employed in Experiment 1. All experimental stimuli, excluding the trial stimuli related to the Animal Stroop task, were identical to Experiment 1. Stimuli for the Animal Stroop task consisted of 36 colored pictures of animals, taken from Bryce et al. (2011). There were three categories of animals based on their real-life size, namely small (e.g. butterfly), medium (e.g. sheep) and large (e.g. rhinoceros). All possible pairs (i.e. small-medium, small-large, medium-large) were presented in equal proportion and the inclusion of medium-sized animals ensured participants could not simply learn the members of the large group and bypass making a comparison between the two animals. In each trial, two different animal images were presented, one positioned on the left and one on the right side of the screen, with a distance from the center measuring 40% of the height of the window (see Fig. 1G and H). One animal stimulus was presented in a small size on the screen (15% of the window’s height) and the other in a large size on the screen (40% of the window’s height). The trial stimuli for the Animal Simon task were the ones used in the Animal Simon task described in Experiment 1.

#### Procedure

The conflict tasks employed in this experiment were the Animal Stroop task and the Animal Simon task. The procedure was identical to that of Experiment 1, except that the Animal Stroop task was presented in the place of the Arrow Simon task. The Animal Stroop task involved the presentation of two colored pictures of animals simultaneously on the left and right side of the screen. One animal was always depicted as larger on screen and the task was to identify which animal would be larger in real-life (relevant dimension) ignoring the size with which they appeared on screen (irrelevant dimension).

Participants gave their response by clicking on the grey box above the relevant animal. In Fig. 1 an example of a congruent and incongruent condition of the Animal Stroop task is depicted (see Fig. 1G and H, respectively). In a congruent trial, the animal that was larger (smaller) in real-life was presented larger (smaller) on the screen compared to the smaller (larger) in real-life animal. In an incongruent trial, the animal that was larger (smaller) in real-life was presented smaller (larger) on the screen compared to the smaller (larger) in real-life animal.

The experimental design and structure were identical to those of Experiment 1, as were the dependent and independent variables. On each trial, relevant animal image(s) were randomly selected without direct repetition from pools of small, medium or big animals for the Animal Stroop task, and from a pool of right- or left-facing animals for the Animal Simon task. There were 12 unique trial types in the Animal Stroop task, comprised of four in which a big and a small (in real-life) animal were presented, four in which a big and a medium animal were presented, and four in which a medium and a small animal were presented. Within each set of four, the side on which the larger in real-life animal was presented and the presentation size of the larger in real-life animal were factorially combined. As described for Experiment 1, the Animal Simon task had four unique trial types. Within a block of 48 trials, each unique Animal Stroop trial was repeated 4 times and presented in random order and for the Animal Simon task, each unique Animal Simon trial was repeated 12 times and presented in random order.

#### Data analysis

##### Data exclusion

As in Experiment 1, all datasets were analyzed as all participants completed the experiment with accuracy levels higher than 60%. The first trial of each block as well as practice trials were not analyzed. For analysis of accuracy, trials in which the previous trial was an error were excluded (2.09%) as were trials with extremely long ITIs (0.05%). For analysis of MT, IT and MAD, additionally to previous exclusions, trials in which the current trial was an error were excluded (2.07%). Extremely long MTs, that is > 3 SDs above the mean (calculated per participant and per experimental condition), were also excluded (1.75%).

##### Planned analyses

Similar to Experiment 1, data from the single Animal Simon blocks were analyzed to test for the presence of within-task Animal Simon CSE, data from the single Animal Stroop blocks were analyzed for the within-task Animal Stroop CSE and data from the alternating blocks were analyzed to test for two across-task CSEs (Animal Stroop-Animal Simon and Animal Simon-Animal Stroop). The presence of within-task and across-task conflict adaptation was evaluated following the same procedure as that in Experiment 1 and 2.

##### Results and discussion

**Fig. 4:**
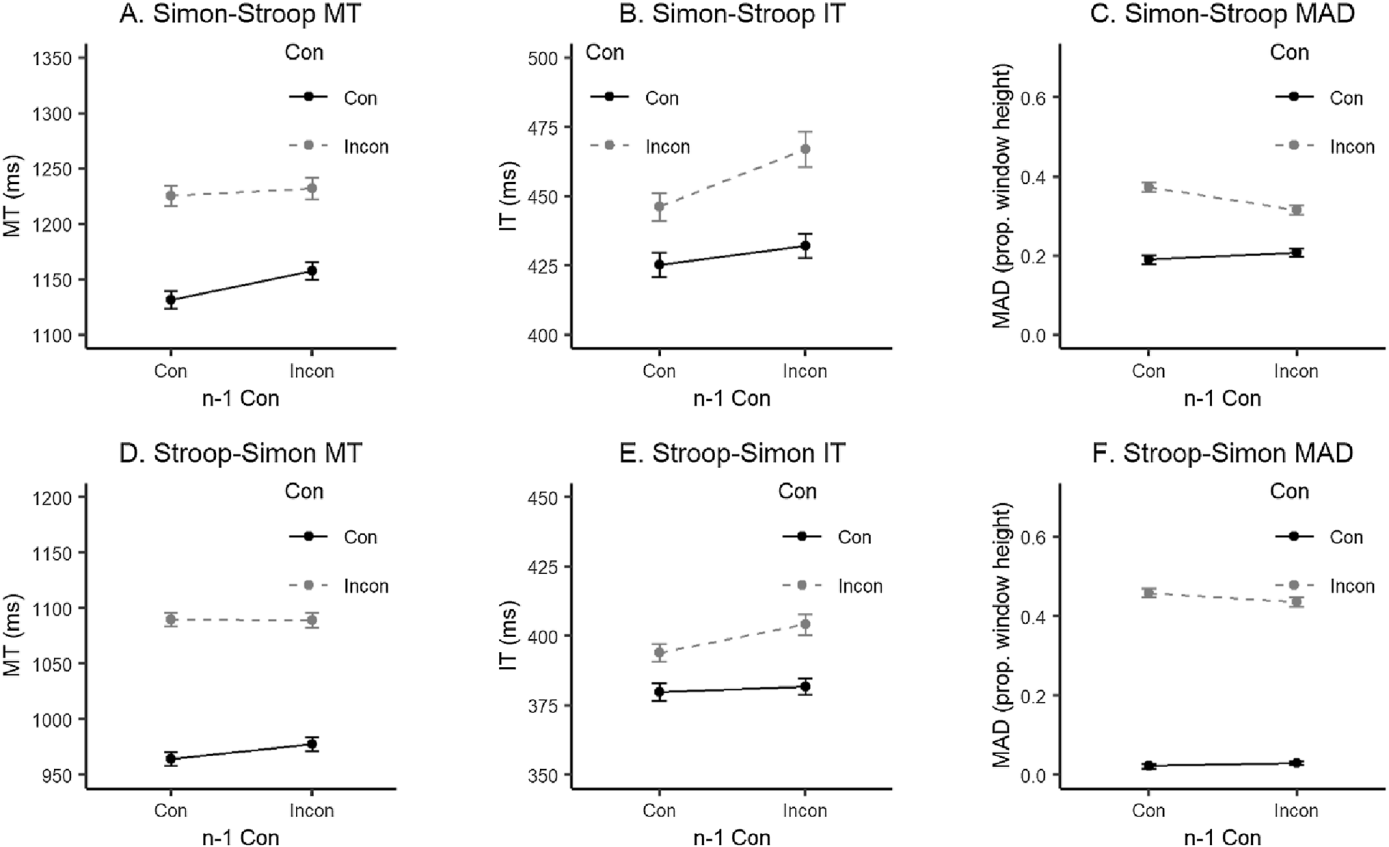
Across-task mean MT, IT and MAD for Experiment 3. In the upper panels (A, B, C) are the results for Animal Simon trials followed by Animal Stroop trials, in the lower panels (D, E, F) are the results for Animal Stroop trials followed by Animal Simon trials. *MT* movement time, *IT* initiation time, *MAD* maximum absolute deviation, *Con* congruency, *n−1* *Con* previous trial’s congruency. Error bars represent within-subject standard errors (using the method from Morey, 2008). Responses were overall slower when the current task was the Animal Stroop task, reflected in different absolute values in y-axes for the MT and IT plots

Statistical results from the repeated measures ANOVAs conducted on accuracy, MT, IT and MAD for both within-task and across-task analyses can be found in Table 3. With regards to within-task effects (plots of which can be found in Supplementary Materials, Fig. S3) a CE was observed in the MT, IT, and MAD in the Animal Simon task. A within-task CSE was also observed in MT and MAD. In the Animal Stroop task, a CE was observed in all measures and a CSE in Accuracy, MT and MAD values. Although the Animal Stroop is the only task in this series with two stimuli in each trial, the within-task results correspond well to those obtained in the Animal Simon, which supports the comparability of the two tasks despite this perceptual difference. For the across-task results (see Fig. 4), in both cases of Animal Simon trials being followed by Animal Stroop trials, and of Animal Stroop trials being followed by Animal Simon trials, a CE was found in all measures and a CSE only in MAD values.

**Table 3:** Two-way ANOVA statistics for Experiment 3 study variables

Variable	Effect					
	Con		n−1 Con		Con × n−1 Con	
	*F*(1, 29)	*η*_p_^2^	*F*(1, 29)	*η*_p_^2^	*F*(1, 29)	*η*_p_^2^
Animal Simon within-task results						
ACC	3.03	0.09	0.04	< 0.01	1.51	0.05
MT	83.66***	0.74	0.23	0.01	31.49***	0.52
IT	6.85*	0.19	4.94*	0.15	0.06	< 0.01
MAD	111.16***	0.79	7.91**	0.21	21.28***	0.42
Animal Stroop within-task results						
ACC	31.50***	0.52	8.01**	0.22	4.49*	0.13
MT	89.14***	0.75	16.66***	0.36	12.63**	0.30
IT	25.84***	0.47	7.12*	0.20	0.88	0.03
MAD	118.36***	0.80	3.57	0.11	6.16*	0.18
Animal Simon–Animal Stroop across-task results						
ACC	12.60**	0.30	0.07	< 0.01	1.09	0.04
MT	46.57***	0.62	4.79*	0.14	1.38	0.05
IT	46.99***	0.62	6.42*	0.18	2.34	0.07
MAD	56.00***	0.66	3.56	0.11	16.04***	0.36
Animal Stroop–Animal Simon across-task results						
ACC	25.27***	0.47	0.55	0.02	0.12	< 0.01
MT	157.90***	0.84	2.18	0.07	0.50	0.02
IT	21.45***	0.43	3.97	0.12	2.34	0.07
MAD	163.70***	0.85	0.75	0.03	4.77*	0.14

*N* = 30. *ANOVA* analysis of variance, *ACC* accuracy, *MT* movement time, *MAD* maximum absolute deviation, *IT* initiation time, *Con* congruency, *n−1* *Con *previous trial’s congruency

*p < 0.05

**p < 0.01

***p < 0.001

In Experiment 3, the two alternating tasks did not share instructions, relevant dimension, irrelevant dimension, or stimulus sets. As mentioned previously, the model that served as a basis for our hypotheses predicts across-task CSEs in the case of highly similar and highly dissimilar contexts. The aim of Experiment 3 was to create highly dissimilar contexts that were expected to yield significant across-task CSEs. This hypothesis was supported only by MAD values, with evidence of conflict adaptation effects in both task orders. In the case of MTs only a main effect of congruency was observed. Similar to Experiments 1 and 2, main effects of congruency and previous trial’s congruency were also present for the mouse-tracking measure IT in support of Erb’s proposal (Erb et al., 2016).

As previously, task order was further investigated in a follow up ANOVA including task order (Animal Simon–Animal Stroop vs. Animal Stroop–Animal Simon) as a factor. In this experiment, the three-way interaction reached significance only for MAD, *F*(1, 29) = 5.49, *p* = 0.026, *η*_p_^2^ = 0.16.

## General discussion

The goal of this online mouse-tracking study was to investigate the different contexts across which cognitive control can be recruited and exerted. More specifically, we investigated whether conflict adaptation, as measured by the CSE, can transfer within and across conflict tasks that differ in the extent to which the relevant and irrelevant dimensions overlap. Participants responded via computer mouse and the measures accuracy, movement time, initiation time and maximum absolute deviation were collected and analyzed within each and across different combinations of adapted Simon and Stroop tasks. Our hypotheses for the presence of across-task conflict adaptation in each of the three experiments were inspired by a review by Braem et al., (2014) where it was proposed that across-task CSEs are more likely to be observed when the two tasks’ context similarity can be characterized as either very high (Experiment 1) or very low (Experiment 3) compared to cases of intermediate or partial similarity (Experiment 2). Overall, the results delivered mixed support for our hypotheses with fairly reliable within-task conflict adaptation, but more varied evidence regarding the transfer of conflict adaptation across different tasks (see Table 4 for a summary of the experimental features and results).

**Table 4:** Summary of experiment features and CSE results from Experiments 1 to 3

Exp	Task		Common dimensions		Within-task CSE		Across-task CSE	
	A	B	Relevant	Irrelevant	A	B	A-B	B-A
1	Animal Simon	Arrow Simon	Yes	Yes	x MT	✓ MT	x MT	✓ MT
					x IT	x IT	x IT	x IT
					✓ MAD	✓ MAD	✓ MAD	✓ MAD
2	Size Simon	Arrow Simon	No	Yes	✓ MT	✓ MT	x MT	✓ MT
					x IT	x IT	✓ IT	x IT
					✓ MAD	✓ MAD	x MAD	✓ MAD
3	Animal Simon	Animal Stroop	No	No	✓ MT	✓ MT	x MT	x MT
					x IT	x IT	x IT	x IT
					✓ MAD	✓ MAD	✓ MAD	✓ MAD

*Exp*. Experiment; *CSE* congruency sequence effect; *MT* movement time; *IT* initiation time; *MAD* maximum absolute deviation. The presence of a significant CSE is indicated by a check mark (*p* < 0.05) or a cross (*p* > / = 0.05)

Let us first consider the across-task data pattern from movement times and maximum absolute deviation values. It was hypothesized that both very high and very low similarity conditions would enable the transfer of conflict adaptation effects; the movement time data were consistent with this in the high similarity condition (Experiment 1) but not in the low similarity condition (Experiment 3). Moreover, and inconsistent with our hypothesis, an across-task CSE in movement times was also reflected in one task order in the medium similarity condition (Experiment 2) where the task features only partially overlapped. Interestingly, the maximum absolute deviation values sometimes provided a different picture. That is, trajectory measures seemed to capture transfer effects across all three experiments and most task orders (with the exception of Size Simon-Arrow Simon in Experiment 2). Taken together, it can be concluded that in all three experiments, conflict experienced in a prior different task influenced response selection processes in the subsequent trial to some extent, and as such our results are not consistent with the hypothesis regarding across-task conflict adaptation.

The time taken to initiate a response movement, reflected in our initiation time measure, was mostly affected by the current and previous trial’s congruency. This is consistent with Erb et al’s (2016) interpretation of initiation time reflecting a response threshold adjustment process resulting in generalized inhibition of all motor output. While Erb et al. (2016) studied these measures within the context of a single task at a time, here we have replicated those results within a single task and extended the finding to the context of alternating tasks. Surprisingly, however, initiation time seems to have also captured an across-task CSE in Experiment 2, in the case of Size Simon trials followed by Arrow Simon trials. What is more, in that task order there was no evidence of conflict adaptation in the maximum absolute deviation values. As mentioned previously, one could speculate a trade-off between the processes captured by the two measures. This may call into question the interpretation of initiation time as reflecting a Response Threshold Adjustment process, as it suggests that the time taken to initiate a movement can also be influenced by the particular combination of current and previous trial’s congruency. Even though the majority of across-task conflict adaptation effects observed here were found primarily in trajectory measures (i.e. MAD values) and not time measures (e.g. IT), a fact that could suggest the characterization of CSEs as a measure involved mainly in the controlled selection stage of processing; future research that directly investigates cases in which time but not trajectory measures reflect conflict adaptation effects could shed more light on the source, locus and processes underlying conflict adaptation.

One reason that our results do not fully support Braem et al.’s (2014) model could be that our experiments did not encompass the entire spectrum of context similarity. More specifically, our classification of the task combinations in Experiments 2 and 3 as medium and low similarity, respectively, could be challenged. Braem and colleagues’ examples of low similarity^1^ task combinations include a sentence processing task combined with a color Stroop task and a gender flanker task combined with a letter flanker task. While on the surface these task pairings may appear more dissimilar than those we selected, we do consider them comparable to our Experiment 3. We postulated that the task combination we selected in Experiment 3 is low in similarity, since the two tasks (Animal Simon and Animal Stroop) differ in terms of the source of conflict as well as memory demands. More specifically, in the Animal Stroop task long-term memory regarding the animals’ real-life sizes is required to resolve the conflict, which is in direct contrast with the Animal Simon task where the information causing conflict as well as the information needed to resolve it is immediately available visually in every trial. Furthermore, it is still unresolved whether the conflict adaptation effects found in the studies listed by Braem in this category (e.g., Kan et al., 2013) may be restricted only to very specific types of language-processing conflicts since other efforts have not replicated those results (e.g., Dudschig, 2022; Simi et al., 2022).

In composing task combinations in the current study, we classified context similarity based on shared relevant and irrelevant dimensions across the tasks. However, this could be too simplistic and perhaps more attention should be given to some additional concepts such as that of task space, the exact source of conflict and how conflict is resolved in each task. According to Xiong and Proctor (2018), task space, task set and relations between stimulus and response (S-R relations) are distinct terms with task set associated with task-relevant information, S-R relations associated with mappings between stimulus and response and task space transcending both task set and S-R relations and including task irrelevant information. Especially in complex situations, task space seems like a promising multidimensional candidate to facilitate the definition of task context borders. With respect to sources of conflict, in his review Egner (2008) notes that sources of conflict can be shared across distinct tasks and that even in the case of distinct sources of conflict, conflict types may still converge at later stages of processing, such as the behavioral output. Interestingly, our mouse-tracking results could be considered consistent with this idea, as sequence effects appear sometimes earlier and sometimes later in the processing stream. All in all, it is clear that the concept of context similarity is still to be precisely defined and should be further addressed in future studies. For now, we can conclude that the occurrence of across-task conflict adaptation cannot be attributed to a simplistic distinction based on shared relevant and irrelevant dimensions as we adopted here.

Another possible reason that our results do not fully support Braem and colleagues’ model could be related to response methods; in the studies reviewed by Braem et al. button responses were employed, whereas we employed mouse-tracking. It should be noted, that even though reaction times as collected through button presses cannot fully map onto movement times as measured through mouse-tracking, Braem et al.’s proposed model had not restricted its application to a specific response method. Our findings, however, can also trigger the question of whether the response mode employed in conflict tasks could directly affect conflict processing and response strategy. It is commonly observed that with hand-tracking response methods, error rates are greatly reduced since there is the opportunity to correct a response (as reflected in the maximum absolute deviation values) before its completion, resulting in a higher number of trials available for data analysis compared to button-response methods. As such, arguably more of the trials on which the most conflict is experienced remain in the dataset when responses are given via hand-tracking methods compared to when responses are given via button presses. To directly investigate whether there are differences in conflict processing depending on the response method it would be interesting to compare different response methods within the context of a single task or combination of tasks. Such studies could help us understand the impact of these design decisions on conflict processing.

Another design decision that may influence the transfer of conflict adaptation effects across tasks is the sequence in which tasks are presented, namely in a predictable or unpredictable manner. When reviewing conflict adaptation studies that employed either alternating or randomly intermixed designs, no notable pattern could be discerned with respect to the presence of transfer effects. It has, however, been suggested that task predictability may lead to more salient task boundaries and therefore less transfer of conflict adaptation effects (Hazeltine et al., 2011). In addition to context similarity (Braem et al., 2014), task predictability may be a further important influence on across-task conflict adaptation that is certainly deserving of further investigation.

An unanticipated and to our knowledge novel finding in this study, was the potential effect of task order on across-task conflict adaptation, which adds to the complexity of conflict processing. This complexity could entail now not only the type or source of conflict of the current task and the congruency of the previous task but also the type or source of conflict of the previous task. Interestingly, additional tests revealed that this trend could not have been driven by differences in the size of the congruency effect. For example, conflict adaptation effects across tasks were observed consistently in both movement times and maximum absolute deviation values when Arrow Simon trials were followed by either Animal Simon (Experiment 1) or Size Simon trials (Experiment 2). However, only in Experiment 2 was there a larger congruency effect in the Arrow Simon task than the other task, as calculated from movement times in single blocks (Experiment 1: Arrow Simon *η*_p_^2^ = 0.48, Animal Simon *η*_p_^2^ = 0.66; Experiment 2: Arrow Simon *η*_p_^2^ = 0.74, Size Simon *η*_p_^2^ = 0.56). Perhaps the task order effect on across-task conflict adaptation is attributable to the different time courses of automatic versus controlled processes in each task impacting the degree to which effects transfer to the other task. To develop a full picture, additional studies applying modelling approaches such as the Diffusion Model for Conflict tasks (Ulrich et al., 2015) could address some of these interpretations or provide further insights into other mechanisms involved.

Attention should additionally be drawn to the fact that task performance and hence the presence or absence of behavioral effects, depend on which task representations are formed, assuming multiple representations can correspond to one task (Kleinsorge, 2021). It has even been demonstrated that this can depend on how the tasks are presented to the participants, through for example instructions (Kleinsorge, 1999). In the current study, we kept the type of instruction constant across all three experiments (tasks were introduced separately and participants were informed they would sometimes be presented within the same block). As such, this cannot be an explanation for our varied findings. Nevertheless, the representations every participant forms about each task separately and in combination with another task cannot always be manipulated with precision by the experimenter, leaving the question of whether two tasks are perceived as separate entities or as one single task unresolved in most circumstances. Clarifying the definition of context similarity and task representation seems to be an important next step for this field of research. There are different possible approaches to tackle this question, such as reviewing the already large literature on CSEs or designing new experiments to manipulate different candidate features of context similarity and identify crucial ones. The empirical experimental approach taken here is just one attempt.

It is of note that the novel tasks developed and employed in this online study elicited all the hallmarks of conflict tasks (CE, within-task CSEs). With respect to confound minimization even though there was no direct repetition of stimuli, since this study employed mouse-tracking and aimed at studying conflict processing children as young as 6 years old, other confounds such as response repetition and lateralization were not controlled for. We are not aware of mouse-tracking studies with conflict tasks that also circumvent the problem of response repetition and lateralization. Indeed, given the nature of the core mouse-tracking measures (such as trajectory) that are of interest in these studies, the options for more complex designs with multiple stimulus–response mappings are limited. It is, however, an important limitation to acknowledge due to concerns about the interpretation of CSEs in designs that are not confound-minimized. Nevertheless, there seems to be consensus in the field that while confounds may enhance CSEs, these effects are still observed in fully confound minimized designs (e.g. Koob et al., 2023). Moving forward, an important aim for future research is to develop confound minimized designs that can be implemented for mouse-tracking and child studies without compromising the translational value of the findings.

In summary, conflict adaptation effects within each of our novel tasks were consistently observed across all experiments, while the transfer of these effects across the different task combinations varied depending on measurement. That is, time measures provided only partial support for our hypothesis and trajectory measures reflected transfer effects across all experiments independent of task combination. This heterogeneity of findings across different measures that capture various aspects of the unfolding decision-making process underlines the importance of applying more sensitive measurement tools to evaluate conflict adaptation. Furthermore, while it is true that variety in findings with respect to across-task transfers is not new to this line of literature, the systematic variation of across-task similarity within one study and the addition of mouse-tracking measurements is. In order to disentangle the meaning and underlying mechanisms of these effects, the conditions under which such transfers occur have to be identified and these conditions need to be addressed more systematically within and across different studies. The current evidence shows that previous conflict affects subsequent conflict processing in all three task combinations, which could reflect a remarkable degree of flexibility of cognitive control in adapting to highly variable and changing environments.

### Supplementary Information

Below is the link to the electronic supplementary material.

Supplementary file1 (DOCX 414 KB)

## Data Availability

This study’s preregistration document, stimuli and data for Experiments 1, 2 & 3 can be found here, respectively: https://osf.io/v573g/, https://osf.io/jaunp/, https://osf.io/pmhqg/.

## Footnotes

1 Note, they use the label “high dissimilarity” where we use “low similarity”.
