# Supplementary material for: How flexible is cognitive control? (Mouse) tracking conflict adaptation across context similarities
Source: Psychol Res. 2023 Sep 28;88(2):562–79. doi: 10.1007/s00426-023-01874-0 (PMC10858099; doi:10.1007/s00426-023-01874-0)
Supplement: Supplementary file 1 — Supplementary file1 (DOCX 414 KB) [file 426_2023_1874_MOESM1_ESM.docx]

**Supplementary Materials**

# Figure S1

*Within-task Mean MT, IT and MAD for Experiment 1.*


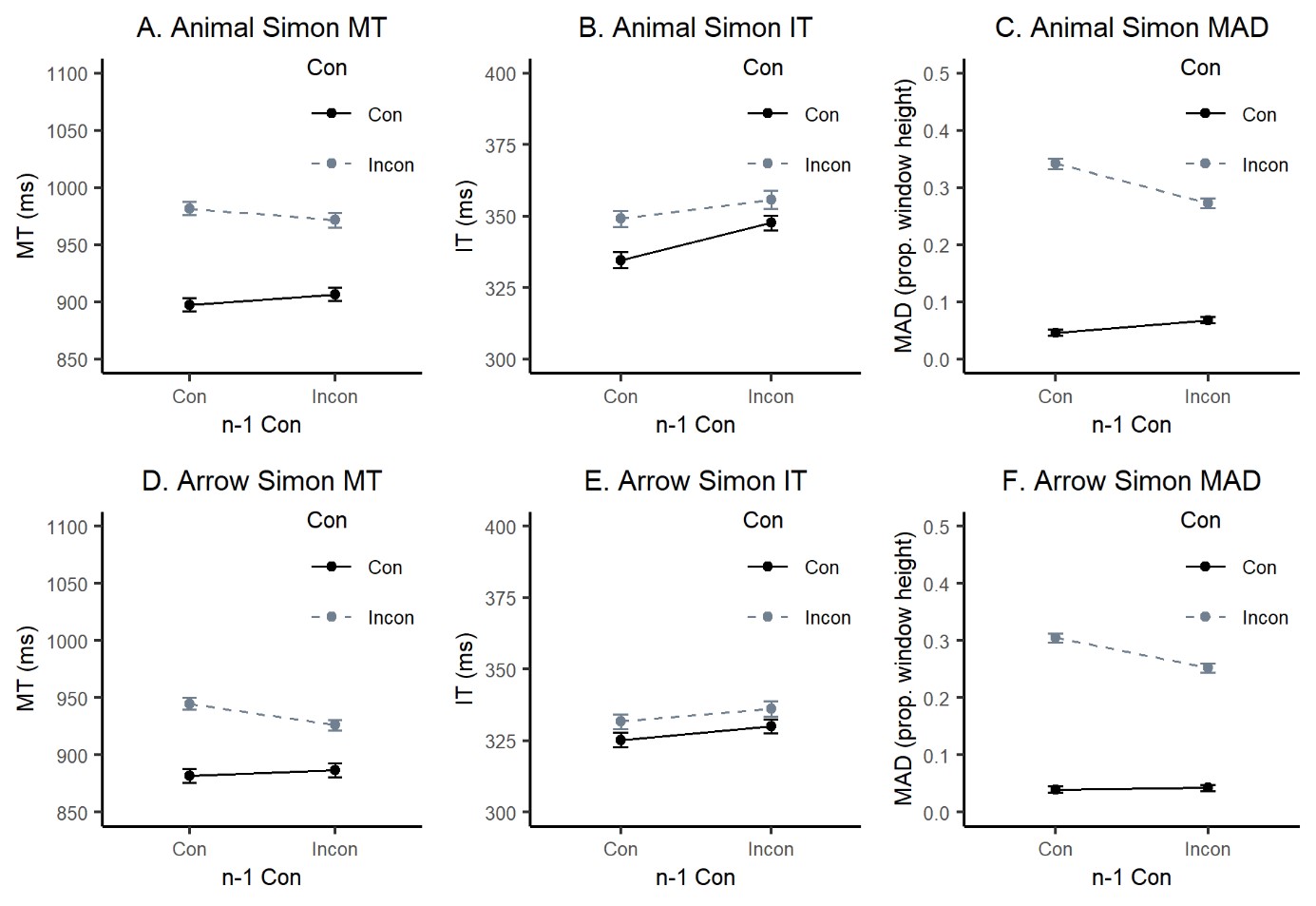


*Note.* MT = Movement Time; IT = Initiation Time; MAD = Maximum Absolute Deviation; Con

= congruency; n-1 Con = previous trial’s congruency. Error bars represent within-subject standard errors (using the method from Morey, 2008).

# Figure S2

*Within-task Mean MT, IT and MAD for Experiment 2.*


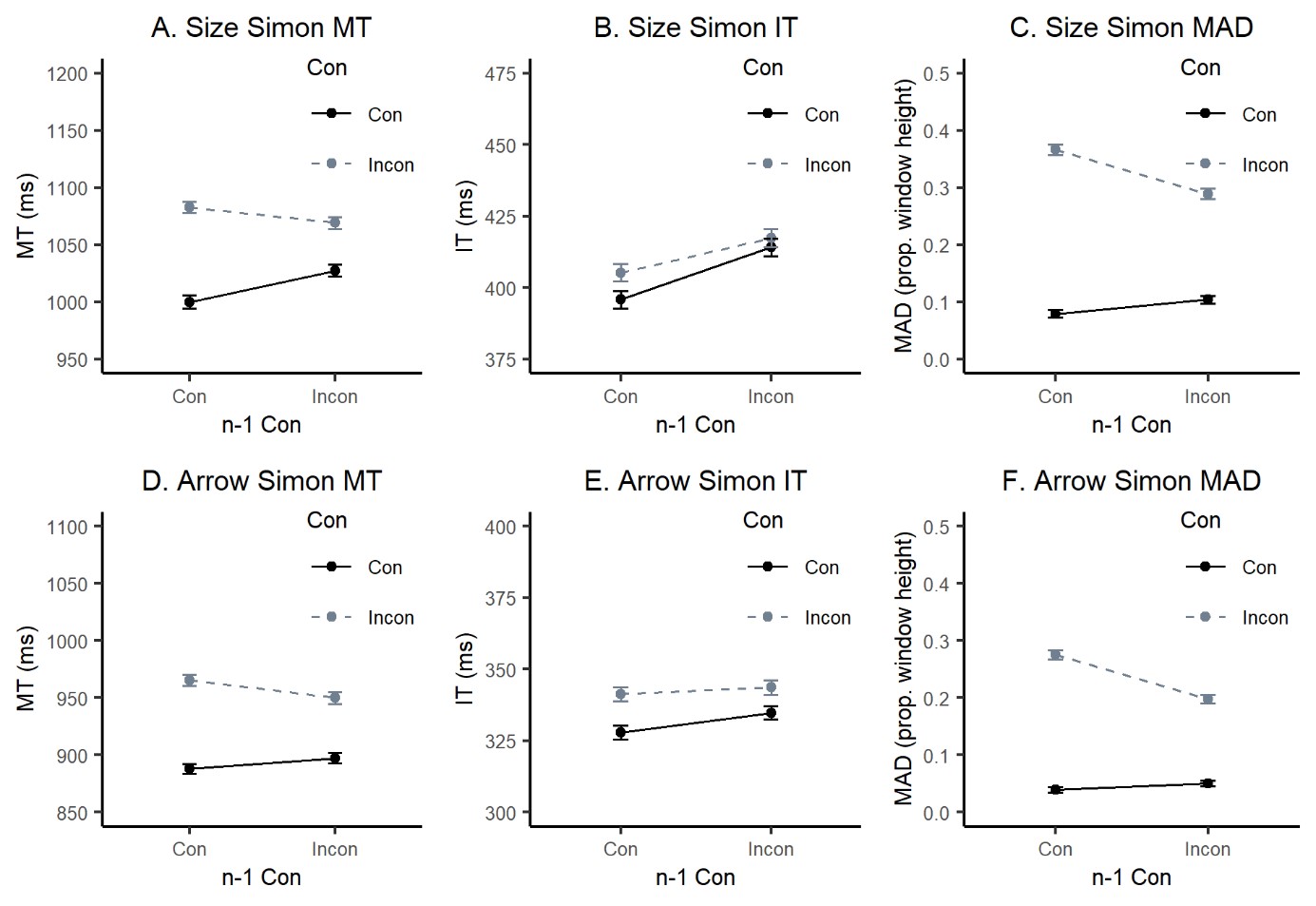


*Note*. MT = Movement Time; IT = Initiation Time; MAD = Maximum Absolute Deviation; Con

= congruency; n-1 Con = previous trial’s congruency. Error bars represent within-subject standard errors (using the method from Morey, 2008). Responses were overall slower in Size Simon task, reflected in different absolute values in y-axes.

# Figure S3

*Within-task Mean MT, IT and MAD for Experiment 3.*


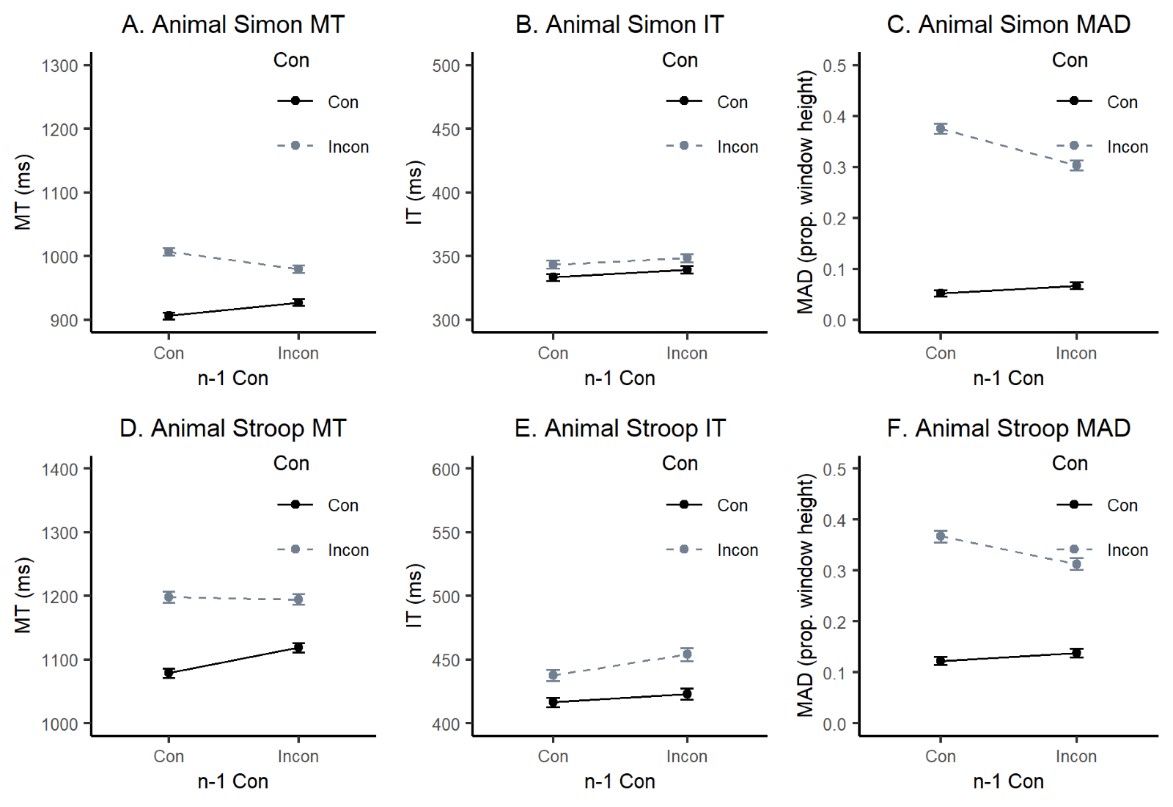


*Note*. MT = Movement Time; IT = Initiation Time; MAD = Maximum Absolute Deviation; Con

= congruency; n-1 Con = previous trial’s congruency. Error bars represent within-subject standard errors (using the method from Morey, 2008). Responses were overall slower in Animal

Stroop task, reflected in different absolute values in y-axes.
